# When the outcome is compositional: A method for conducting compositional response linear mixed models for physical activity, sedentary behaviour and sleep research

**DOI:** 10.1371/journal.pone.0340373

**Published:** 2026-01-28

**Authors:** Aaron Miatke, Ty Stanford, Tim Olds, Francois Fraysse, Carol Maher, Josep Antoni Martin-Fernandez, Dot Dumuid

**Affiliations:** 1 Alliance for Research in Exercise, Nutrition and Activity, Allied Health and Human Performance, University of South Australia, Adelaide, SA, Australia,; 2 Centre for Adolescent Health, Murdoch Children’s Research Institute, Melbourne, Australia; 3 Department of Computer Science, Applied Mathematics and Statistics, University of Girona, Girona, Spain; Central Queensland University, AUSTRALIA

## Abstract

Time use is compositional in nature because time spent in sleep, sedentary behaviour and physical activity will always sum to 24 h/day meaning any increase in one behaviour will necessarily displace time spent in another behaviour(s). Given the link between time use and health, and its modifiable nature, public health campaigns often aim to change the way people allocate their time. However, relatively few studies have investigated how movement-behaviour compositions change longitudinally (with repeated measures), due to experimental design elements (e.g., intervention effects), or differences due to participant socio-demographic characteristics (e.g., sex, socio-economic status) within clustered sampling designs. This may be because most mixed-model packages that account for the random effects do not natively support a multivariate outcome such as movement-behaviour composition. In the current paper we provide a practical framework of how to implement a compositional multivariate-response linear mixed model that can be used to model the entire 24h movement-behaviour composition as the dependent variable within a multilevel framework. The method accounts for covariances across and within response variables at the grouping (individual, cluster etc.) and covariance between response variables at the observation level. Results are therefore invariant to the chosen log-ratio basis used to construct the response variables (i.e., mathematically equivalent models). The method outlined is applicable to many designs including longitudinal cohort studies, intervention trials, and clustered cross-sectional designs (e.g., students within schools, patients within clinics). In a worked example we show how this approach can be used to investigate how time is reallocated in children across the school year.

## 1.0 Introduction

Time spent in daily movement behaviours (sleep, sedentary behaviour (SB), physical activity) has been linked with many health measures, ranging from adiposity to mental health, cognition and mortality risk [1]; largely investigated in cross-sectional or observational data. Due to its modifiable nature, intervention and health promotion efforts have long attempted to improve specific aspects of time use, such as increasing time spent in physical activity [2,3], reducing sedentary time [4,5], or improving sleep [6]. Most statistical analyses of the effectiveness of such interventions consider only one activity in isolation, even though the constant sum constraint of a 24-h day means that other activity(ies) will necessarily undergo compensatory changes. As all the activities are important to health outcomes, they should all be considered when planning and executing movement behaviour interventions [7]. Understanding shifts in time use resulting from natural temporal (e.g., circadian and circannual) cycles and life transitions are also important considerations. It is imperative to be able to include the full 24-h composition of movement behaviours in the same statistical model when assessing the effectiveness of interventions. The inclusion of all raw 24-h movement behaviour variables (min/day) in statistical models has been problematic due to their constant sum constraint and inherent perfect multi-collinearity which may produce spurious results [8]. This has been overcome through the application of compositional data analysis (CoDA), whereby 24-h compositions are expressed as logratios prior to their inclusion in statistical models [9].

While CoDA is now commonly applied when 24-h movement-behaviour compositions are considered as independent or predictor variables, fewer studies have used CoDA to consider movement behaviour compositions as dependent or response variables [10]. There is currently no accepted methodology for analysing movement behaviour compositions in a multi-level framework that could be used to, for example, assess whether mean compositions change over time with repeated measurements on individuals, if there are intervention or experimental group effects in compositions over time, or if compositions are associated with sociodemographic or environmental factors while accounting for clustered sampling designs. As such, researchers have generally used other approaches to investigate changes or group differences in compositions. Most have ignored the compositional nature of the data entirely and modelled minutes per day of each behaviour separately [11,12]. Some have tried to respect the compositional nature of their data and expressed the compositions as a set of orthonormal log-ratio (olr) coordinates which are iteratively used as the dependent variables in multiple univariate models while accounting for the random effects of repeated measures on participants (as outlined further in section 1.2). Others have investigated how compositions change over time by creating a ‘change composition’ via perturbation. This reduces the data to a single observation and eliminates the need to model random effects. These are all useful approaches, however, they also all have drawbacks. The first approach ignores the compositional nature of the data completely; the second relies on interpreting individual olr coordinates which limited practical meaning; and the third lacks flexibility in its application. For example, it requires complete data and can only be used with two timepoints.

This paper is structured as follows: in Section 1, we provide background on the rationale for CoDA for 24-h movement behaviours. Section 1.1 introduces the log-ratio methodology and how it is applied within regression modelling, and Section 1.2 describes previous frequentist approaches to analysing compositions as the dependent variables, noting the inability of these methods to include multivariate response variables when the data have a multi-level structure. In Section 2, we present a method for a compositional multivariate-response linear mixed model (CMRLMM). Section 3 provides an example of the application of our CMRLMM to real-world data of Australian school children’s movement behaviour patterns across various time points. We also compare the CMRLMM to both the univariate response compositional linear mixed model and to the non-compositional linear mixed model fitted separately for each behaviour in raw minutes/day (e.g., Sleep, SB, light physical activity [LPA], moderate-to-vigorous physical activity [MVPA]). These comparators reflect common practice in the literature and illustrate the consequences of ignoring the correlations between olr coordinates/behaviours and the constant-sum constraint. In Section 4, we provide a concluding discussion on strengths and weaknesses of the new method, and areas for future development and research.

### 1.1 Brief overview of CoDA and application to 24-h movement behaviours

CoDA is used in behavioural epidemiology to analyse time spent across 24-hour movement behaviours, including sleep, SB, and physical activity. The log-ratio methodology transforms raw compositional data into orthonormal log-ratio (olr) coordinates (also known as isometric log-ratio coordinates [ilr]) before their inclusion in compositional regression models. However, current methods struggle to address a common issue in epidemiological studies: the multi-level structure of movement behaviour data caused by repeated measurements or clustered sampling designs.

Indeed, a composition 𝐮 is a vector in $R_{+}^{D}$ where the only relevant information is contained in the ratios between its components, these components of the composition are aptly named *compositional parts*. In the context of time-use epidemiology, these compositional parts typically represent the time spent in sleep, SB, LPA and MVPA. For example, the ratio of time spent in SB to time asleep within a 24-h window is invariant to whether those times are expressed as proportions of the day, hours or minutes and have specific properties due to the sample space. However, it is also worth noting that it is possible to conceptualise a time-use composition in a variety of ways. Instead of creating a time-use composition based on activity intensities, it is also possible to divide the day into time spent in contextual domains such as work, commuting, household activities, leisure etc. [13]. Compositions also exist in other fields within the behavioural and health sciences such as dietary macronutrient compositions (carbohydrate, fat, protein) [14] and body composition (fat, muscle, bone) [15]. In all these examples, a D-part composition can be expressed as a vector $𝐱=[x_{\text{1}},x_{\text{2}},\ldots ,x_{D}]$, where all parts are non-negative (but ideally strictly positive) and sum to a positive constant κ. Typically, its sample space is mathematically written as

$S^{D}=\left\{𝐱\;|\;𝐱\in R^{D},\;\sum_{i=\text{1}}^{D}x_{i}=\kappa ,x_{i}>\text{0}\right\}\;$ where $S^{D}$ is known as the *D*-part simplex or *D*-simplex, a (D−1) dimensional subset of real space $R^{D}$ [16]. Because compositions only contain relative information, a composition 𝐮 can be closed to any positive constant such that


$$\begin{array}{c} 𝐱=\left[x_{\text{1}},x_{\text{2}},\ldots ,x_{D}\right]=C\left[u_{\text{1}},u_{\text{2}},\ldots ,u_{D}\right]=\left[\frac{\kappa \cdot u_{\text{1}}}{\sum_{i=\text{1}}^{D}u_{i}},\frac{\kappa \cdot u_{\text{2}}}{\sum_{i=\text{1}}^{D}u_{i}},\ldots ,\frac{\kappa \cdot u_{D}}{\sum_{i=\text{1}}^{D}u_{i}}\right] \end{array}$$ (1)

where C is the closure operation to the constant κ. For example, if the parts of the vector represent time spent in the behaviours sleep, SB, LPA and MVPA, the composition could equivalently be closed to κ = 1440 (min/day), κ =24 (hour/day), κ =1 (proportion of day). When considering differences, or changes, in compositions, traditional algebraic addition and subtraction operations for Euclidean space, are not suitable as they are not scale invariant nor sub-compositionally coherent [17,18]. However, in the simplex space compositions can be perturbed such that $𝐱⊕𝐲=C\left[x_{\text{1}}y_{\text{1}},x_{\text{2}}y_{\text{2}},\ldots ,x_{D}y_{D}\right]$ which is analogous to addition in real space. In addition, the powering of $𝐱\in {\text{S}}^{D}$ by a constant α∈R, $\alpha ⊙𝐱=C\left[x_{\text{1}}^{\alpha },x_{\text{2}}^{\alpha },\ldots ,x_{D}^{\alpha }\right]$, which plays the typical role of the product of a vector by a scalar. Using these operations a linear regression model with compositional response can be formulated as


$$\begin{array}{c} \hat{𝐱}=\beta_{0}⊕\left(\;\text{t}⊙\beta_{1}\right) \end{array}$$ (2)

where the coefficients $\beta_{0},\beta_{1}\in {\text{S}}^{D}$ and t is a real covariate. Further, the neutral element 1 =C[1,…, 1] = [k/D, k/D, ..., k/D]  has the expected properties such that 𝐱 ⊕ C[1,…, 1]= C[1,…, 1]⊕𝐱 = 𝐱. Finally the inverse element of **x**, is defined as $C[\text{1}/x_{\text{1}},\;\text{1}/x_{\text{2}},\;...,\;\text{1}/x_{D}]$, denoted as ⊖𝐱. It can be shown that when a composition is perturbed by its inverse it will result in the neutral element such that 𝐱⊕(⊖𝐱= (⊖𝐱)⊕𝐱 = C[1,…, 1]. While ‘stay in the simplex’ algebra like that outlined above can be used to summarise or interpret CoDa, most multivariate statistical techniques are not suitable for raw CoDa and require expressing the raw data in terms of log-ratios of the parts. As such, most compositional techniques used in time-use epidemiology favour using the log-ratio approach, specifically the olr transformation [9]. While other log-ratio approaches have been proposed, such as the additive log-ratio (alr) or centred log-ratio (clr), these have drawbacks that limit their use within statistical models such as mixed models. Namely, alr coordinates are asymmetric, meaning that distances are not preserved within the simplex space. This means results depend on the compositional part chosen as the denominator when constructing the coordinates. Unlike alr coordinates, clr coordinates are isometric, meaning distances are preserved. However, the clr coordinates are also restrictive and spurious when used within statistical models because of the zero-sum constraint [9,19,20]. Neither of these issues exist when using the olr transformation which is generally preferred in most modelling applications, including within time-use epidemiology [9]. The olr transformation involves expressing D-part compositional vector of movement behaviour data that exist in the simplex, $𝐱=\left[x_{\text{1}},x_{\text{2}},\ldots ,x_{D}\right]\in S^{D}$, into D-1 olr-coordinates [21,22] that exist in real space, $𝐳=\text{olr}(𝐱)=[z_{\text{1}},z_{\text{2}},\ldots ,z_{D-\text{1}}]\in R^{D-\text{1}}$. That is, the vector 𝐳 are the coordinates of composition 𝐱 in an olr-basis. Importantly, these olr-coordinates can be used in standard multivariate statistical models [23]. For example, the linear regression model in Eq. (2) is expressed in olr-coordinates as $\text{olr}(\hat{𝐱})={\text{olr}(\beta }_{0})+\text{t}\cdot \text{olr}(\beta_{1})$.

An olr-basis can be created using a data-driven method such as Principal Balances [24] or R-mode cluster analysis [25]. Alternatively, the knowledge of the researcher can be used to improve the interpretation of the models when creating the olr-basis by a sequential binary partition (SBP) process, which is generally preferred in time-use research [15]. An SBP process uses a (D – 1) x D dimensional sign matrix to iteratively divide the compositional parts until all groups consist of a single component [26].

The olr-coordinates of the general form can be defined as


$$\begin{array}{c} z_{j}=\sqrt{\frac{p_{j}.n_{j}}{p_{j}+n_{j}}}\text{ln}\frac{{{(x}_{k_{\text{1}}}\;\cdot \cdot \cdot \;\;x_{k_{p_{j}}})}^{\text{1}/p_{j}}}{{{(x}_{l_{\text{1}}}\;\cdot \cdot \cdot \;\;x_{l_{n_{j}}})}^{\text{1}/n_{j}}},j=\text{1},\ldots ,D-\text{1} \end{array}$$ (3)

where the $p_{j}$ and $n_{j}$ are the number of parts in the *j-*th row of the sign matrix that are coded positive and negative, respectively and $k_{\text{1}},\ldots ,k_{p_{j}}$ are the indexes of parts in the numerator and $l_{\text{1}},\ldots ,l_{n_{j}}$ are the indexes of parts in the denominator of each row of the sign matrix [26]. Note that the olr-coordinate in Equation (3) is a “balance” between the average of two sets of parts. Balance coordinates may be of use when a researcher is interested in distinct groups of behaviours [27]. In the context of 24-hour movement behaviours, this means that each olr-coordinate represents a contrast between groups of behaviours such as sleep vs. waking activities, or active vs. passive behaviours. Together, these coordinates fully describe how time is distributed across the day while preserving the relative nature of the data. If a researcher has a particular interest in one behaviour, they may use pivot coordinates of the general form


$$\begin{array}{c} z_{j}=\sqrt{\frac{D-j}{D-j+\text{1}}}\text{ln}\left(\frac{x_{j}}{{{(x}_{j+\text{1}}\;\cdot \cdot \cdot \;\;x_{D})}^{\text{1}/(D-j)}}\right),j=\text{1},\ldots ,D-\text{1} \end{array}$$ (4)

Here, the first olr-coordinate ($z_{\text{1}})$ reflects the dominance of one behaviour ($x_{\text{1}}$) relative to the geometric mean of the remaining behaviours and is of use when a researcher has a particular interest in one behaviour. Note that $z_{\text{1}}=\sqrt{\frac{D}{D-\text{1}}}\cdot \text{ln}\left(\frac{x_{\text{1}}}{\sqrt[{D}]{\prod_{k=\text{1}}^{D}x_{k}}}\right)$, that is, the first olr-coordinate is proportional to the first clr-coordinate The remaining olr-coordinates, $z_{\text{2}},\ldots ,z_{D-\text{1}}$, are then created in a similar manner with the denominator of the previous coordinate split with until no parts remain [28].

While hypothesis-driven construction of olr-coordinates may provide one way of interpreting findings from compositional models, there are limitations to how meaningful these contrasts are to everyday life. For example, the first pivot coordinate ($z_{\text{1}}$) must be interpreted as the relative increase of one activity, while the geometric mean of the remaining activities is reduced. In real life, changes in the geometric mean of a group of activities may be difficult to conceptualise, it is often easier to interpret findings within the simplex space [29]. Importantly, while the interpretation for individual olr-coordinates will change depending on the basis chosen when creating the olr-coordinates, collectively they are equivalent and will retain all relative information about the movement behaviour composition no matter how they are constructed such that $𝐱={\text{olr}}^{-\text{1}}\left(𝐳\right)$ for any olr-basis. This means in order to make inferences about the movement behaviour composition as a whole, the vector of olr-coordinates $𝐳=[z_{\text{1}},z_{\text{2}},\ldots ,z_{D-\text{1}}]$ must be considered collectively. One option is to use the statistical model to compute point estimates for scenarios of interest, relevant to the research question. For example, if the logratios are considered as predictors of a health outcome in a linear regression model, the model parameters can be used to estimate the value of the health outcome for a selection of different movement behaviour compositions that emulate the reallocation of time between activities [30]. If the logratios are considered as the dependent variables in a multivariate response linear regression model, the model parameters can be used to estimate what the logratios (and the corresponding 24-h movement-behaviour compositions) would be at different levels/values of the predictor or independent variable. However, when considering compositional responses, standard multivariate response linear regression models are often unsuitable in epidemiological studies due to non-independence of observations resulting from repeated measurements on sampling units (e.g., participants; or clustered sampling designs, e.g., participants within health centres). In these instances, a linear mixed-effects model (LMM) that extends the general linear model to include both fixed and random effects that account for correlated observations is a popular and understood method. Importantly, commonly used statistical software packages for LMMs within a frequentist framework (STATA, SPSS, SAS, R) do not support the inclusion of multivariate outcomes natively (without some data manipulation and model re-specification), such as movement behaviour composition expressed as logratios [31–33]. This difficulty explains why an approach previously, as described in Section 1.2, to investigate longitudinal changes in movement-behaviour composition has been to use multiple univariate response LMMs, each with a different olr-coordinate as the outcome [34–38].

### 1.2 Compositional univariate-response linear mixed model

Previous studies investigating changes in movement-behaviour compositions with repeated measurements on the same individuals (i = 1,2,…,N) over T timepoints $t_{i\text{1}},\;t_{i\text{2}},\;...,\;t_{iT}$ have used a univariate compositional LMM of the form outlined in Equation (5). This model will have time as the level 1 response which is nested within individuals as the level 2 response and has been used by some researchers in the general form


$$\begin{array}{c} z_{ij}\;=\;\beta_{\text{0}}\;+\beta_{\text{1}}t_{ij}+\;b_{\text{0}i}\;+\;\epsilon_{ij},\;i=\text{1},\text{2},\ldots ,N;j=\text{1},\text{2},\ldots ,T; \end{array}$$ (5)

$$b_{\text{0}i}\sim N\left(\text{0},\;\sigma^{\text{2}}\right),\;\;\epsilon_{ij}\sim N\left(\text{0},\;\tau^{\text{2}}\right)$$

Where $z_{ij}$ is the response for an individual olr-coordinate, for the *i-*th subject at the *j-*th timepoint, $\beta_{\text{0}}$ is the mean value for z when all predictors are equal to zero, $b_{\text{0}i}$ is the random intercept for *i-*th subject, $\beta_{\text{1}}$ is the slope coefficient representing the mean linear slope change over time, timepoint $t_{ij}$ is the specific *j*-th time for subject *i*, and $\epsilon_{ij}$is the residual error. In the context of 24-h movement-behaviour research, this type of model has typically been used to assess whether time spent in specific behaviours, or balances of behaviours, changes across repeated measurement occasions (e.g., baseline, 6 months, and 12 months), or differs between intervention and control groups. For example, a researcher may use Equation (5) to test whether the relative balance between active and sedentary time changes following a lifestyle intervention or investigate average trajectories in time-use composition in a longitudinal cohort study. Previously, researchers have generally investigated compositional outcomes within a multilevel framework using multiple models of the general form Equation (5) in one of three ways. Approach 1) has involved iteratively constructing D sets of pivot coordinates (Equation (4)), with $z_{\text{1}}$ reflecting the dominance of a different behaviour in each instance and using this coordinate as the dependent variable in D different LMM analyses. For example, this approach has been used previously to investigate intervention effects on 24-h movement behaviours by fitting a separate model where $z_{\text{1}}$ corresponds to the pivot coordinate for sleep, then for SB, LPA, and MVPA, respectively [34,35]. Alternatively, approach 2) similarly involves a ‘multiple models’ approach for each component in the vector of olr-coordinates constructed using a single basis [36–38], that is, a separate model for each element of the vector $𝐳=\left[z_{\text{1}},z_{\text{2}},\ldots ,z_{D-\text{1}}\right]$ (Equation (3)) where each coordinate represents a particular balance of behaviours, for example, active vs passive behaviours. Approach 3) involves ignoring the compositional nature of the data and fitting separate models of a similar form to Equation (3) that treat each behaviour as an independent outcome. That is, fit Equation (3) with raw minutes/day of each behaviour as the dependent variable. While this third approach is strictly not a compositional model, it is still the probably the most used approach currently by applied time use researchers, so we include it here for completeness.

These methods can be useful approaches; however, they also have limitations. Firstly, all three approaches may have an inflated type 1 error rate associated with running repeated analyses with the same data [39]. Moreover, while approach 1 may be useful if a researcher has a particular interest in a single behaviour, as described earlier the coefficients for a single pivot coordinate are often practically less meaningful to interpret [40]. Similar difficulties exist when interpreting results for individual olr coordinates using approach 2, particularly for coordinates $\left[z_{\text{2}},\ldots ,z_{D-\text{1}}\right]$, where only some behaviours will be involved in their creation. Approach 2 may allow for results to be transformed back into the simplex to allow for more meaningful interpretations via model-based estimates. However, this requires estimates from multiple, independent models to be pieced together. While approach 3 is widely used, it disregards the structural linear dependence among compositional behaviours (e.g., guaranteed structural spurious non-zero correlation [41]. By treating each behaviour as independent, individually regressing behaviours in a univariate manner and cannot ensure that predicted time across behaviours sum to 24 hours (see Supplementary file for examples). Importantly, while approach 2 does model each element of the complete vector of olr coordinates, it ignores any correlation structure among the olr-variables. By using D−1 individual models of form Equation (5), we are assuming that the random effects and errors come from D−1 separate, and unrelated, normal distributions. However, this is unlikely to be true in the case of CoDa, where the olr-coordinates are intrinsically multivariate in nature and are usually correlated. From an interpretation standpoint, this means our unrelated models provide no information on how behavioural trade-offs may occur to fit into the 24-h day, for example to see whether people who sleep more also accumulate relatively more PA within their waking day. Importantly, using the multiple models approach also means that once transformed back to the simplex, model-based estimates and residuals may differ depending on the sign matrix and associated olr-basis used to construct the coordinates (see examples in supplementary material 1). This is a problem when working with compositional data, where invariance under change of olr basis is one of the fundamental principles [23]. Indeed, the use of multiple, independent models as outlined in approach 2 above implicitly assumes no relationships between olr coordinates. This is equivalent to specifying a diagonal matrix (heterogenous variance with zero correlation between elements) in the multivariate sense. However, if, after the change of basis, the model is solved again using the multiple-univariate model procedure outlined in approach 2, it is implicitly assumed that the matrices are diagonal, and therefore, the same result as before the change of basis cannot be obtained. Simply put, the only way to obtain equivalent results before and after the change of basis is by using a multivariate procedure that accounts for the relationship between olr coordinates (see supplementary file for further details).

Moreover, while it is possible to test the effects on individual olr-coordinates using the multiple-models approach, there is difficulty in performing a single test of the joint effects on the complete vector of olr-coordinates [42], for example, to explore whether the composition of 24-h movement behaviours changed differently between intervention and control groups. In order to overcome these limitations a CMRLMM is required, as presented in Section 2.

## 2. Compositional multivariate-response linear mixed model

We propose extending the univariate mixed model above in Equation (5) to the multivariate case where response vectors are **not** modelled with implied independence. By taking the univariate equation above and adding subscript r to indicate the specific olr-response in question (i.e., r=1, 2, ..., D−1), the dependence between response vectors can be more easily specified. Our multivariate formula now becomes:


$$\begin{array}{c} Z_{rij}\;=\;\beta_{\text{0r}}\;+\beta_{\text{1r}}t_{rij}+\;b_{\text{0}ri}\;+\;\epsilon_{rij} \end{array}$$ (6)

where $Z_{rij}$ is response r for the ith participant at the jth timepoint; $\beta_{\text{0}r}$ is the fixed intercept specific to response olr-coordinate; $\beta_{\text{1}r}$ is the fixed ‘slope’/contrast between time-point j and time-point 1 specific to response olr-coordinate r. Note, the general formula outlined here assumes a linear change over timepoints and is used for simplicity in notation. In instances where this is not true, $\beta_{\text{1r}}t_{rij}$ can be replaced to estimate olr-responses over the T timepoints via an indicator function as follows $\sum_{j^{*}=\text{2}}^{T}\;\text{I}(j^{*}=j\{\beta {\}}_{\text{1}rj},$ where I(a) is an indicator function that is equal to 1 when the argument a is true, 0 otherwise. So far, this appears similar to the previous formula. However, unlike Equation (5), in Equation (6), the random effects and error will be of length D−1, and are now assumed to come from a single multivariate normal distribution, rather than multiple unrelated univariate distributions, as follows:


$${𝐛}_{\text{0}i}=(\begin{array}{c} b_{\text{0}\text{1}i}\\ b_{\text{0}\text{2}i}\\ \vdots \\ b_{\text{0}(D-\text{1})i} \end{array})\;\sim MVN\left(0_{D-1},\;𝐆\right)$$

where ${𝐛}_{\text{0}ri}$ are potentially differently varying and correlated random intercepts for each response olr-coordinate r, specific to person i; and


$$\epsilon_{ij}=\;(\begin{array}{c} \varepsilon_{\text{1}ij}\\ \varepsilon_{\text{2}ij}\\ \vdots \\ \varepsilon_{(D-\text{1})ij} \end{array})\;\sim MVN\left(0_{D-1},\;𝐑\right)$$

where $\epsilon_{rij}$ are potentially differently varying and correlated random errors for each response olr-coordinate r, specific to person i at time-point j for each response olr-coordinate r; with group- and residual-side variance and covariance (D−1)×(D−1 matrices


$$𝐆=\left[\rho_{ij}^{\left(g\right)}\sigma_{i}\sigma_{j}\right],\;\;i,j=\text{1},\text{2},\ldots ,D-\text{1}\;$$ (7)

and


$$𝐄=\left[\rho_{ij}^{\left(e\right)}\tau_{i}\tau_{j}\right],\;\;i,j=\text{1},\text{2},\ldots ,D-\text{1}$$ (8)

Respectively. Or, written more fully in expanded form


$$\begin{array}{c} 𝐆=[\begin{array}{cccc} \sigma_{\text{1}}^{\text{2}} & \rho_{\text{1}\text{2}}^{\left(g\right)}\sigma_{\text{1}}\sigma_{\text{2}} & \cdots & \rho_{\text{1}(D-\text{1})}^{\left(g\right)}\sigma_{\text{1}}\sigma_{\left(D-\text{1}\right)}\\ & \sigma_{\text{2}}^{\text{2}} & \cdots & \rho_{\text{2}(D-\text{1})}^{\left(g\right)}\sigma_{\text{2}}\sigma_{\left(D-\text{1}\right)}\\ & & \ddots & \vdots \\ & & & \sigma_{\left(D-\text{1}\right)}^{\text{2}} \end{array}] \end{array}$$ (9)

and


$$\begin{array}{c} 𝐄=[\begin{array}{cccc} \tau_{\text{1}}^{\text{2}} & \rho_{\text{12}}^{\left(e\right)}\tau_{\text{1}}\tau_{\text{2}} & \cdots & \rho_{\text{1}(D-\text{1})}^{\left(e\right)}\tau_{\text{1}}\tau_{\left(D-\text{1}\right)}\\ & \tau_{\text{2}}^{\text{2}} & \cdots & \rho_{\text{2}(D-\text{1})}^{\left(e\right)}\tau_{\text{2}}\tau_{\left(D-\text{1}\right)}\\ & & \ddots & \vdots \\ & & & \tau_{\left(D-\text{1}\right)}^{\text{2}} \end{array}], \end{array}$$ (10)

where correlations ρ at the differing levels of model are differentiated with the (g) and (e) superscripts, where $\rho_{ii}^{\left(g\right)}=\rho_{ii}^{\left(e\right)}=\text{1}$. Note that both matrices are symmetrical, that is, ${𝐆}_{ij}={𝐆}_{ji}$ and ${𝐄}_{ij}={𝐄}_{ji}$ for any (ij) entry.

Unlike in Equation (5), this model accounts for the correlation between each olr-coordinate by estimating covariances between random effect and residual error terms as shown in the off-diagonal elements above which are constrained to be equal to zero when using the multiple models approach outlined earlier. Thus, the model outlined in Equation (6) allows olr-responses to flexibly vary (and covary) for each olr at both the group (participant) level and residual level. Estimates can then be made for olr-coordinates while respecting the multivariate nature of the data. Just like in the model outlined in Equation (5) the estimates at all levels of the model, including the residuals, will be specific to the basis chosen when constructing the olr-coordinates. However, when (uniquely) transformed back in the simplex space using the inverse transformation $\text{ol}{\text{r}}^{-\text{1}}\left(\beta \right)$, the compositional coefficients, and any time-use estimates made using the model, are invariant to the basis used to construct the original olr-coordinates. Currently, in a frequentist framework, the multivariate response linear mixed model of Equation (6) cannot be fitted in R in the most popular linear mixed model packages nlme [43] and lme4 [44]. However, there are two potential solutions to this problem: (a) use a Bayesian framework to fit the multivariate response linear mixed model. For example, using brms::brm(); or (b) re-define the data structure to fit an equivalently specified (frequentist) univariate response linear mixed model using nlme::lme() and lme4::lmer(). Each paradigm has advantages and limitations. Bayesian methods are highly flexible, can naturally accommodate multivariate outcomes, and provide full posterior distributions of parameters for richer uncertainty quantification. However, they are typically more computationally demanding and require appropriate prior specification. Frequentist approaches, by contrast, enable formal hypothesis testing and are computationally efficient in some scenarios. However, problems can sometimes arise when fitting models with complex random-effects structures, where convergence or boundary fit issues may occur. Specifically, in the case of (near) zero variance components that cause boundary fit issues, Bayesian estimation can offer greater numerical stability through proper priors of the variance parameters where sampling at 0 poses no issues. In practice, the frequentist framework (knowingly or unknowingly) is also currently the most familiar statistical approach used by time-use and other CoDA researchers. The popularity of frequentist methods has motivated the formalisation of this approach in the hope to create a template for future compositional response linear multilevel models for repeated observations or clustered data as (although choosing one approach should not be mutually exclusive of the other). For the interested reader or CoDA practitioner, Bayesian CMRLMMs can be fit natively on “wide format” (discussed below) multivariate responses data using the R package `multilevelcoda` that also contains a discussion of Frequentist vs Bayesian approaches [45].

In order to be able to fit Equation (6) using the frequentist framework, the data have to be restructured so that all olr-coordinate responses are in a single column to be used as the dependent variable. We term the re-defined data structuring to fit an equivalently specified univariate response linear mixed model the “stacked response” linear mixed model approach. This terminology is borrowed from the limited online resources [46,47], and even fewer published descriptions [48,49], and of course how the multivariate response vectors are stacked to make a univariate response vector. Additional dummy variables are then needed to specify the olr-coordinate response in question, that is, a dummy variable for each response r = 1,2,…,D-1. Our original dataset which was of length *ij* (*i* individuals over *j* timepoints each), now becomes length *ijr* with one row per olr-coordinate, per participant, per timepoint. We can then specify a single model that allows for individual changes in each olr-response, while accounting the structure of the random effects and error terms as specified in Equations (9) and (10). An example of the stacking process is outlined below for r=1,2,3 in Tables 1 and 2. In the restructured dataset the vector of olr-coordinates is contained in a single column along with the dummy variables

**Table 1:** The original data in general form in wide format for the *i*th individual.

Olr-response vector			Predictor
z_1i1_	z_2i1_	z_3i1_	t_i1_
z_1i2_	z_2i2_	z_3i2_	t_i2_
z_1i3_	z_2i3_	z_3i3_	t_i3_
z_1i4_	z_2i4_	z_3i4_	t_i4_
…	…	…	…

**Table 2:** The restructured dataset in general form in long format for the *i*th individual after ‘stacking’ the multivariate olr-response variable.

Olr value	olr1/Response 1 indicator dummy variable ($\delta_{\text{1}}$)	olr2/Response 2 indicator dummy variable ($\delta_{\text{2}}$)	olr3/Response 3 indicator dummy variable ($\delta_{\text{3}}$)	predictors
z_1i1_	1	0	0	t_i1_
z_2i1_	0	1	0	t_i1_
z_3i1_	0	0	1	t_i1_
z_1i2_	1	0	0	t_i2_
z_2i2_	0	1	0	t_i2_
z_3i2_	0	0	1	t_i2_
z_1i3_	1	0	0	t_i3_
z_2i3_	0	1	0	t_i3_
z_3i3_	0	0	1	t_i3_
z_1i4_	1	0	0	t_i4_
z_2i4_	0	1	0	t_i4_
z_3i4_	0	0	1	t_i4_
…	…	…	…	…

The dummy variables are binary variables representing which olr-response is being considered. By expanding Equation (5) we can now fit Equation (8) where the response is the notionally ‘univariate’ response associated with the stacked vectors of responses


$$z_{ij}=\;\delta_{\text{1}}[\beta_{\text{0}\text{1}}\;+\beta_{\text{1}\text{1}}t_{ij}+\;b_{\text{0}\text{1}i}\;+\;\epsilon_{\text{1}ij}+\;\delta_{\text{2}}\left[\beta_{\text{0}\text{2}}\;+\beta_{\text{1}\text{2}}t_{ij}+\;b_{\text{02}i}\;+\;\epsilon_{\text{2}ij}\right]\;+\;...\;+\;\delta_{D-\text{1}}\left[\beta_{\text{0}(D-\text{1})}\;+\beta_{\text{1}(D-\text{1})}t_{ij}+\;b_{\text{0}(\text{D}-\text{1})\text{i}}\;+\;\epsilon_{(D-\text{1})ij}\right]\;\;$$ (11)

Or more succinctly expressed as


$$z_{rij}=\;\sum_{k=\text{1}}^{D-\text{1}}\delta_{k}\beta_{\text{0}k}+\sum_{k=\text{1}}^{D-\text{1}}\delta_{k}\beta_{\text{1k}}t_{ij}+\sum_{k=\text{1}}^{D-\text{1}}\delta_{k}b_{\text{0k}i}+\sum_{k=\text{1}}^{D-\text{1}}\delta_{k}\epsilon_{rij},\;\text{r}=\text{1},\text{2},\ldots ,\text{D}-\text{1}.$$ (12)

Where $\delta_{\text{1}},\;\delta_{\text{2}},...,\;\delta_{D-\text{1}}$, are the olr (or response index) indicator variables associated with each olr-response as specified in Table 2 where the value of $\delta_{r}$ = 1 for the rows of data containing the dependent variable r, and 0 otherwise. Thus, the dummy variables act as an additional level to the model which simply defines the response structure. Of note, when compared to the univariate mixed model in Equation (5), the multivariate model in this form also no longer has an overall intercept. Instead, the intercept for each olr-response is estimated separately based on the dummy coding used. Equation (11) can now be used to estimate the vector of responses simultaneously while respecting the multivariate nature of the data. Expanding the formula to include additional demographic predictors such as socio-economic status, body-mass index etc. is then relatively simple. These can be included in the model call by including interaction terms between the dummy coding used and variables of interest to allow each fixed effect to have a specific response (index) associated estimate.

### Multivariate test on fixed effects

Another key advantage of the CMRLMM is the ability to perform a multivariate test on the fixed effects, such as a multivariate F test or Wald chi-square test to test for significance in changes in composition across timepoints, between groups, or other variables of interest. When using the multiple models approach outlined in Section 1.2, it is only possible to perform univariate tests on individual olr-coordinates. However, when using a CMRLMM, it is also possible to conduct tests that are multivariate in nature because the model includes the complete vector of olr-coordinates ${𝐳}_{ij}=[z_{\text{1}ij},z_{\text{2}ij},\ldots ,z_{(D-\text{1})ij}]$. Moreover, because the covariances between are accounted for at both levels of the model, the compositional representation of the model is equivalent regardless of the basis chosen when constructing the olr-coordinates, meaning results will be consistent.

## 3. Example

In this section we use data from the Life on Holidays (LoH) study, for which a full protocol describing data collection methods has been published previously [50]. LoH was a longitudinal cohort study based in Adelaide, Australia, that aimed to track changes in 24-h activity composition, diet and weight status of primary school-aged children during the school year and summer school holiday periods (n = 241). Ethical approval was obtained from The University of South Australia Human Research Ethics Committee (200980), the South Australian Department of Education and Child Development (2008–0055) and the Adelaide Catholic Education Centre (201820) for the original Life on Holidays study. Time use was measured using wrist-worn GENEActiv accelerometers at five timepoints across two school years between February 1^st^ 2019 – November 30^th^ 2021: Timepoint 1, at the start of Grade 4 (February-March); Timepoint 2, at the end of Grade 4 (October-November); Timepoint 3, during the summer holiday period; Timepoints 4 & 5, at the start and end of the Grade 5 school year, respectively. Time-use composition was conceptualised as a 4-part composition (D = 4) consisting of time spent in sleep, SB, LPA, or MVPA. Each minute of the day was classified as either SB, LPA or MVPA from the accelerometer recordings using validated cutpoints [51] with sleep time distinguished from waking time using a validated algorithm [52]. Unconditional CMRLMM was initially created without the addition of any covariates. Baseline categorical socio-economic status as determined by parental income, and continuous BMI z-score were then included in the full model as an example of how to include time-invariant covariates.

Children’s movement-behaviour compositions at each study time-point (index *j* currently ignored for clarity) were expressed as olr-coordinates using the SBP and sign matrix shown below, where the 4-part compositional vector $𝐱=\left[x_{\text{1}},x_{\text{2}},x_{\text{3}},x_{\text{4}}\right]$, reflected time spent in sleep, SB, LPA and MVPA, respectively. Conceptually, the SBP matrix is a representation of a (divisive) clustering dendrogram starting with the entire set of behaviours and recursively partitioning the (sub)sets of behaviours until each leaf/node contains only a set of two or less behaviours. In the accompanying sign matrix, each row represents an olr coordinate, *+ 1* identifies behaviours in the numerator group of the log ratio, *–1* identifies those in the denominator group of the log ratio, and *0* indicates that the behaviour is not part of that specific olr coordinate. Note: when considering the raw compositional coefficients, time-use estimates and interaction effects for the complete vector of coordinates, the choice of SBP is arbitrary.


$$\begin{array}{c} \begin{array}{ccccc} olr\;coordinate\backslash composition & x_{\text{1}} & x_{\text{2}} & x_{\text{3}} & x_{\text{4}}\\ z_{\text{1}} & +\text{1} & -\text{1} & -\text{1} & -\text{1}\\ z_{\text{2}} & \text{0} & +\text{1} & -\text{1} & -\text{1}\\ z_{\text{3}} & \text{0} & \text{0} & +\text{1} & -\text{1} \end{array} \end{array}$$

According to the sign matrix above the resultant olr-coordinates $𝐳=[z_{\text{1}},z_{\text{2}},z_{\text{3}}]$ were then calculated as follows (ignoring the person *I* at timepoints *j* notation for simplicity), where the first coordinate $z_{\text{1}}$ represents the ratio of sleep to the geometric mean of all waking behaviours combined; the second coordinate, $z_{\text{2}},$ represents the ratio of SB to active behaviours (LPA + MVPA), and the third coordinate, $z_{\text{3}}$, contrasts LPA to MVPA as outlined below.


$$z_{\text{1}}=\sqrt{\frac{\text{3}}{\text{4}}}\text{ln}\frac{Sleep}{{\left(SB\;\cdot \;LPA\;\cdot \;MVPA\right)}^{\text{1}/\text{3}}}$$

$$z_{\text{2}}=\sqrt{\frac{\text{2}}{\text{3}}}\text{ln}\frac{SB}{{\left(LPA\;\cdot \;MVPA\right)}^{\text{1}/\text{2}}}$$

$$z_{\text{3}}=\sqrt{\frac{\text{1}}{\text{2}}}\text{ln}\left(\frac{LPA\;}{MVPA\;}\right)$$

For person i=1, 2,…, 241 and time-point j=1, 2,…, 5, the corresponding three response (r=1,2,3) *olr-coordinates* $z_{rij}$ were then modelled as


$$\begin{array}{c} z_{rij}=\beta_{\text{0}r}+\sum_{j^{*}=\text{2}}^{\text{5}}\;\text{I}\left(j^{*}=j\right)\;\beta_{\text{1}rj}+b_{\text{0}ri}+\in_{rij} \end{array}$$ (12)

where $\beta_{\text{0}r}$ is the fixed intercept specific to response olr-coordinate r; I(a) is the indicator function that is equal to 1 when the argument a is true, 0 otherwise; $\beta_{\text{1}rj}$ is the fixed ‘slope’/contrast between time-point j(=2,3,4,5) and time-point 1 specific to response olr-coordinate r; ${𝐛}_{\text{0}i}=[\begin{array}{c} b_{\text{0}\text{1}i}\\ b_{\text{0}\text{2}i}\\ b_{\text{0}\text{3}i} \end{array}]\sim MVN\left(0_{\text{3}},𝐆\right)$ are potentially differently varying and correlated random intercepts for each response olr-coordinate r, specific to person i; $\in_{ij}=[\begin{array}{c} \in_{\text{1}ij}\\ \in_{\text{2}ij}\\ \in_{\text{3}ij} \end{array}]\sim MVN\left(0_{\text{3}},𝐄\right)$ are potentially differently varying and correlated random errors for each response olr-coordinate r, specific to person i at time-point j; with group- and residual-side variance and covariance matrices respectively defined according Equations (9) and (10).

It is noteworthy that In the LoH study, a linear relationship between the follow-up timepoints and each olr-response cannot be assumed as time use was hypothesised to differ during the school holiday period when compared to the in-school timepoints. Therefore, categorical timepoint indicators, contrast to the first study time point, were used. However in a more general case of J repeated measures (or even $J_{i}$ repeated measures per person i) over time that may not be equally spaced but a linear relationship between the follow-up timepoints and each olr-response, one would replace the somewhat notationally clumsy $\sum_{j^{*}=\text{2}}^{\text{5}}\;\text{I}(j^{*}=j\{\beta {\}}_{\text{1}rj}$ terms simply with $\;\beta_{\text{1}r}t_{ij}$ for each response r where $t_{ij}$ is potentially continuous and unique time value for the jth repeated measure for person i. This principle is equally applicable when time is not the predictor of interest, for example in cross-sectional clustered designs adiposity could be treated as continuous (zBMI) or categorical (overweight/obese status). The choice between categorical or continuous predictors does not alter the core structure of the model. An additional level of nesting to account for nesting of participants within schools was also tested, however the variance components for this level of the model were very low, suggesting little school-to-school variation. The school-level random effects were subsequently dropped in the interest of model parsimony. In order to fit Equation (12), the three response variables were then stacked into a single column to be used as the dependent variable as described earlier. Models were fitted using R package the nlme::lme() [43]. R code along with alternate model specification using the lme4::lmer() package [44] are provided in supplementary material 1.

Table 3 presents parameter estimates and standard errors for the fixed effects of Equation (12). p values are reported using the ‘inner outer’ approximation of degrees of freedom as is default in nlme::lme() [43]. The accurate estimation of degrees of freedom and p values in multilevel models is often discussed [53]. Other degrees of freedom approximation methods have been proposed, including the Satterthwaite and Kenward–Roger approximations [54], along with other methods of quantifying uncertainty (e.g., bootstrapping). However, a detailed comparison of these methods is beyond the scope of this article. Results suggest higher values for all three olr-coordinates over time, in particular for timepoint 3. zBMI was positively associated with $z_{\text{3}}$ representing the balance of LPA to MVPA suggesting the ratio of LPA to MVPA increased as zBMI increased.

**Table 3:** Fixed effect estimates for the CMRLMM.

	z_1_			z_1_			z_1_		
	Estimate (S.E)	t-statistic	p value	Estimate (S.E)	t-statistic	p value	Estimate (S.E)	t-statistic	p value
Intercept	0.83 (0.02)	48.0	<0.01	0.94 (0.04)	22.8	<0.01	0.93 (0.04)	26.2	<0.01
Timepoint (vs. T1)									
T2	0.01 (0.01)	0.7	0.48	0.01 (0.02)	0.49	0.63	−0.01 (0.02)	−0.34	0.74
T3	0.09 (0.01)	6.7	<0.01	0.22 (0.03)	8.5	<0.01	0.21 (0.03)	7.5	<0.01
T4	0.03 (0.01)	2.1	0.03	0.09 (0.02)	3.8	<0.01	0.01 (0.03)	0.4	0.71
T5	0.04 (0.01)	2.9	<0.01	0.12 (0.03)	4.6	<0.01	0.05 (0.03)	1.7	0.09
zBMI	0.01 (0.01)	0.7	0.45	0.003 (0.02)	0.2	0.87	0.05 (0.01)	3.1	<0.01
Income (vs. middle)									
High	−0.001 (0.02)	−0.1	0.96	0.01 (0.05)	0.1	0.92	−0.07 (0.04)	−1.6	0.12
low	0.03 (0.02)	1.4	0.16	0.10 (0.05)	2.1	0.04	0.03 (0.04)	0.8	0.41

Abbreviations: S.E = standard error; zBMI = body mass index z-score (a measure of adiposity).

The estimated level 2 random effects (between individual) variance/covariance matrix as described in Equation (9) are shown below:


$$\hat{𝐆}=[\begin{array}{ccc} {\hat{\sigma }}_{\text{1}}^{\text{2}} & {\hat{\rho }}_{\text{1}\text{2}}^{\left(g\right)}{\hat{\sigma }}_{\text{1}}{\hat{\sigma }}_{\text{2}} & {\hat{\rho }}_{\text{1}\text{3}}^{\left(g\right)}{\hat{\sigma }}_{\text{1}}{\hat{\sigma }}_{\text{3}}\\ & {\hat{\sigma }}_{\text{2}}^{\text{2}} & {\hat{\rho }}_{\text{2}\text{3}}^{\left(g\right)}{\hat{\sigma }}_{\text{2}}{\hat{\sigma }}_{\text{3}}\\ & & {\hat{\sigma }}_{\text{3}}^{\text{2}} \end{array}]=[\begin{array}{ccc} {\hat{\text{0}.\text{1}\text{1}}}^{\text{2}} & {\hat{\text{0}.\text{7}\text{5}}}_{*}{\hat{\text{0}.\text{1}\text{1}}}_{*}\hat{\text{0}.\text{29}} & {\hat{\text{0}.\text{6}\text{4}}}_{*}{\hat{\text{0}.\text{1}\text{1}}}_{*}\hat{\text{0}.\text{22}}\\ & {\hat{\text{0}.\text{29}}}^{\text{2}} & {\hat{\text{0}.\text{4}\text{9}}}_{*}{\hat{\text{0}.\text{2}\text{9}}}_{*}\hat{\text{0}.\text{22}}\\ & & {\hat{\text{0}.\text{22}}}^{\text{2}} \end{array}]=[\begin{array}{ccc} \hat{\text{0}.\text{0}\text{1}\text{2}} & \hat{\text{0}.\text{0}\text{2}\text{4}} & \hat{\text{0}.\text{0}\text{1}\text{6}}\\ & \hat{\text{0}.\text{0}\text{8}\text{2}} & \hat{\text{0}.\text{0}\text{3}\text{2}}\\ & & \hat{\text{0}.\text{0}\text{5}\text{0}} \end{array}]$$

And the estimated level 1 residual (within individual and time-point) error variance/covariance matrix as described in Equation (10):


$$\hat{𝐄}=[\begin{array}{ccc} {\hat{\tau }}_{\text{1}}^{\text{2}} & {\hat{\rho }}_{\text{1}\text{2}}^{\left(e\right)}{\hat{\tau }}_{\text{1}}{\hat{\tau }}_{\text{2}} & {\hat{\rho }}_{\text{1}\text{3}}^{\left(e\right)}{\hat{\tau }}_{\text{1}}{\hat{\tau }}_{\text{3}}\\ & \tau_{\text{2}}^{\text{2}} & {\hat{\rho }}_{\text{2}\text{3}}^{\left(e\right)}{\hat{\tau }}_{\text{2}}{\hat{\tau }}_{\text{3}}\\ & & {\hat{\tau }}_{\text{3}}^{\text{2}} \end{array}]=[\begin{array}{ccc} {\hat{\text{0}.\text{1}\text{2}}}^{\text{2}} & {\hat{\text{0}.\text{6}\text{8}}}_{*}{\hat{\text{0}.\text{1}\text{2}}}_{*}\hat{\text{0}.\text{24}} & {\hat{\text{0}.\text{6}\text{4}}}_{*}{\hat{\text{0}.\text{1}\text{2}}}_{*}\hat{\text{0}.\text{26}}\\ & {\hat{\text{0}.\text{24}}}^{\text{2}} & {\hat{\text{0}.\text{6}\text{4}}}_{*}{\hat{\text{0}.\text{2}\text{4}}}_{*}\hat{\text{0}.\text{26}}\\ & & {\hat{\text{0}.\text{26}}}^{\text{2}} \end{array}]=[\begin{array}{ccc} \hat{\text{0}.\text{0}\text{1}\text{4}} & \hat{\text{0}.\text{0}\text{1}\text{9}} & \hat{\text{0}.\text{0}\text{1}\text{9}}\\ & \hat{\text{0}.\text{0}\text{5}\text{7}} & \hat{\text{0}.\text{0}\text{3}\text{9}}\\ & & \hat{\text{0}.\text{0}\text{6}\text{5}} \end{array}]$$

Positive correlations for all three olr-coordinates were observed in both the within- and between-person covariance matrices. Importantly, these correlations are overlooked when using the multiple univariate response modelling approach outlined earlier. Practically, this impacts our results in multiple ways. Firstly, the covariance components offer additional insight into how people allocate their time and can offer behaviourally meaningful inferences depending on the sign matrix used to construct the coordinates. For example, the positive correlation between olr1 and olr2 in the between-person G matrix suggests that those individuals who on average spend more time in sleep vs awake (olr1), also tend to spend more of their waking day accumulating SB than active time (olr2). These insights are lost when fitting multiple unrelated models to either individual olr coordinates or raw min/day of each behaviour. Likewise, the correlations between the fixed effect parameters allow for valid joint uncertainty for the full olr vector (supplementary material). Additionally, the model fit statistics and statistical power will improve when compared to a multiple models approach used previously. To demonstrate this, we can fit the equivalently specified multivariate response model with the random effect and residual covariance matrices assumed to be diagonal matrices (covariances constrained to zero), referred to as the multivariate unrelated outcomes model. This model will provide the same estimates at all levels of the model as those provided by the multiple univariate models. However, because the multivariate unrelated outcomes model is nested within the fully multivariate model outlined above, it can be compared via a likelihood ratio test. Results of the likelihood ratio test suggest that the addition of the covariances between olr coordinates at both levels of the model improves model fit (Table 4).

**Table 4:** likelihood ratio test comparing CMRLMM to the multivariate unrelated outcomes model with assumed diagonal covariance matrices.

Model	df	AIC	BIC	loglik	L ratio	p value
CMRLMM	36	−1261	−1048	666	(1 vs 2)	
Unrelated model	30	−104	74	82	1169	<0.0001

Abbreviations: CMRLMM = compositional multivariate response linear mixed model; df = degrees of freedom; AIC = Akaike information criterion; BIC = Bayesian information criterion; loglik = log-likelihood.

**Fig 1:**
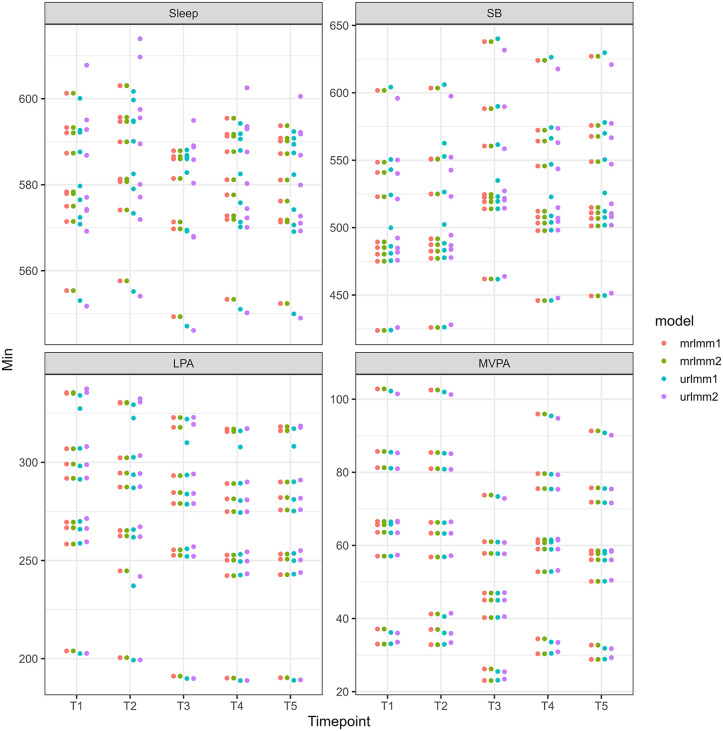
Fitted values for 10 randomly selected level-2 units with coordinates constructed using two different olr bases. Abbreviations: mrlmm = multivariate response linear mixed model; urlmm = univariate response linear mixed model.

Another important difference is that the results for the CMRLMM will be invariant to the basis used to construct the olr-coordinates. This is not the case when constraining the covariances to be diagonal matrices as is the case when fitting independent models on each olr coordinate. To demonstrate this, we can see in Fig 1 the fitted values for 10 randomly sampled level-2 units that have been transformed back to their compositional representation when using the CMRLMM and those from multiple, independent models with olr coordinates that were constructed using two different orthonormal bases. It can be seen that the compositional representation is equivalent for the two CMRLMMs. However, results using the independent models approach differ depending on the basis used to construct the olr coordinates. Importantly, both also differ from those provided from the CMRLMM. Further model comparisons are provided in supplementary file S1, S2, S3 Files.

When compared to fitting four separate and independent non-compositional models on raw minutes/day additional benefits are seen when using the CMRLMM. In addition to providing no information on the relationships between behaviours as described above by not explicitly modelling covariances, the non-compositional models do not respect the constant sum constraint of the data (supplementary file S1, S2, S3 Files).

Unlike the multiple models approach used previously, the multivariate mixed model also allows for a single test of the fixed effects for the joint effect on the vector of olr-coordinates which will be invariant to the basis chosen when constructing the olr-coordinates. The results of the multivariate F-test are presented in Table 5. Using ‘inner-outer’ approximation of denominator degrees of freedom [55], the F statistic for interaction between the vector of olr-coordinates and timepoints (F(12, 2475) = 9.832, p < 0.001) shows that the fixed effects of the model suggest movement-behaviour composition is significantly different across timepoints. Results also suggest that there was a significant interaction between zBMI and movement-behaviour composition (F(3,2475) = 4.44, p = 0.004), whereas parental income and movement-behaviour composition were not significantly associated (F(6,2475) = 1.86, p = 0.08). The ability to perform multivariate tests such as these is limited when creating separate models for individual olr coordinates. Three-way interactions between olr-coordinates, timepoint and covariates were also tested, but were not significant, suggesting children followed a similar pattern of across all timepoints.

**Table 5:** Multivariate F test for the CMRLMM.

	Numerator DF	Denominator DF	F-value	p-value
Z	3	2475	868.9	<0.001
Z:time	12	2475	9.8	<0.001
Z:zBMI	3	2475	4.4	0.004
Z:Income	6	2475	1.9	0.084

While the results of the multivariate F-test suggest movement-behaviour compositions are different across timepoints, they do not give an indication of which timepoints differ, on which components in what direction. In order to investigate this, the estimates presented in Table 3 which are specific to the basis chosen when constructing the olr-coordinates $𝐳=[z_{\text{1}},z_{\text{2}},z_{\text{3}}]$, need to be back-transformed to their corresponding raw compositional representation $𝐱=\left[x_{\text{1}},x_{\text{2}},x_{\text{3}},x_{\text{4}}\right]$, using $\beta_{x}=\text{ol}{\text{r}}^{-\text{1}}\left(\beta_{z}\right)$. For example, back transforming the vector of intercept estimates $olr^{-\text{1}}\left(\left[\hat{\beta_{\text{0}\text{1}}},\hat{\beta_{\text{0}\text{2}}},\hat{\beta_{\text{0}\text{3}}}\right]\right)$, will result in the estimated mean movement-behaviour composition at baseline (assuming zBMI of zero and reference SES group), as below


$$\begin{array}{c} \hat{{𝐱}_{\cdot \text{1}}}=\left[\hat{x_{\text{1}\cdot \text{1}}},\hat{x_{\text{2}\cdot \text{1}}},\hat{x_{\text{3}\cdot \text{1}}},\hat{x_{\text{4}\cdot \text{1}}}\right]=olr^{-\text{1}}\left(\left[\hat{z_{\text{1}\cdot \text{1}}},\hat{z_{\text{2}\cdot \text{1}}},\hat{z_{\text{3}\cdot \text{1}}}\right]\right)=olr^{-\text{1}}\left(\left[\hat{\beta_{\text{0}\text{1}}},\hat{\beta_{\text{0}\text{2}}},\hat{\beta_{\text{0}\text{3}}}\right]\right) \end{array}$$ (13)

Once transformed into the simplex space, the compositional coefficient estimates are now invariant to the basis chosen when constructing the olr-coordinates for both the fixed effects and random effects. After back transforming the estimates for timepoints T2 to T5, the compositional coefficients can be interpreted as the perturbation vector for that timepoint when applied to the baseline composition (which has been closed to 1440 min/day in Table 6) to obtain estimates for a given timepoint. The perturbation vector can be interpreted substantively as relative reallocations for each behaviour when compared to the neutral perturbation vector, subject to closure as outlined in Section 1.1. For example, with a four-part composition, as in our demonstration, the neutral perturbation vector [1/D, …, 1/D] is [0.25, 0.25, 0.25, 0.25]. When the compositional coefficients for timepoints 2–5 are compared to the neutral perturbation vector we can see that in our example dataset time appears to be reallocated away from MVPA (values <0.25) and towards SB (values >0.25) as children age across the timepoints, with the largest changes occurring during T3 (the school holiday period).

**Table 6:** Compositional coefficient estimates for fixed effects.

Timepoint	Sleep	SB	LPA	MVPA
Intercept ${\hat{\beta }}_{0}$	584	483	294	79
T2	0.252	0.251	0.247	0.250
T3	0.266	0.289	0.256	0.190
T4	0.255	0.267	0.240	0.237
T5	0.257	0.271	0.243	0.228

**Fig 2:**
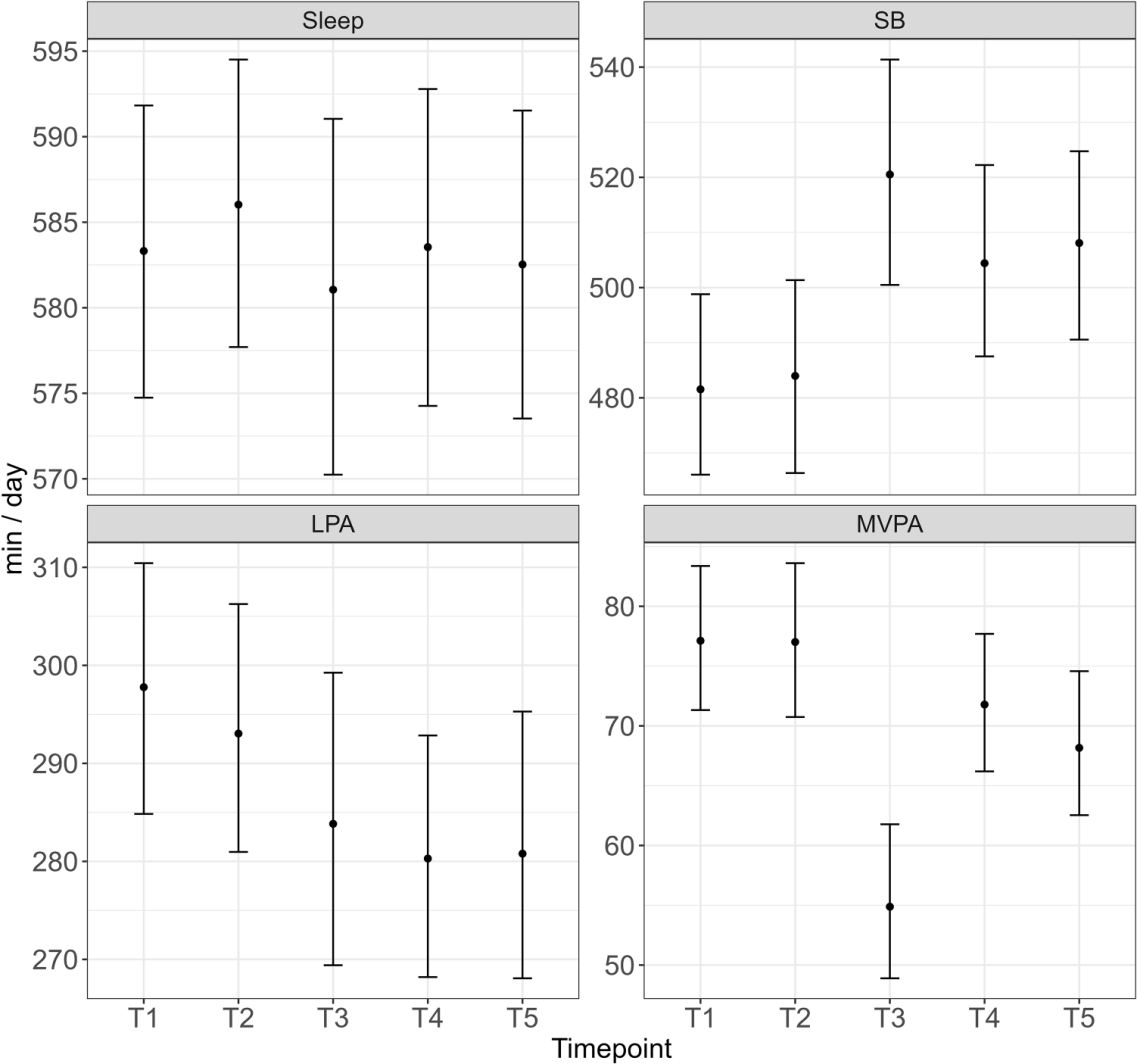
Estimated movement-behaviour compositions for an average participant across the five timepoints. Sleep: range 581-586 min/day. SB: range 481-520 min/day. LPA: range 280-298 min/day. MVPA: range 55-77 min/day.

While compositional coefficients are meaningful, it may be preferred to make model-based point estimates for the compositions, and differences, across timepoints using the fixed effects from the CMRLMM. Fig 2 provides estimated movement-behaviour compositions at the five timepoints (for a participant of mean zBMI and income category) via predicted olr-values that have been back transformed into the simplex space $S^{\text{4}}$, 95% percentile intervals for the compositions were estimated using non-parametric ‘cases’ bootstrapping procedure with 1000 replicates, which resamples level-2 observations (participants) [56,57].

**Fig 3:**
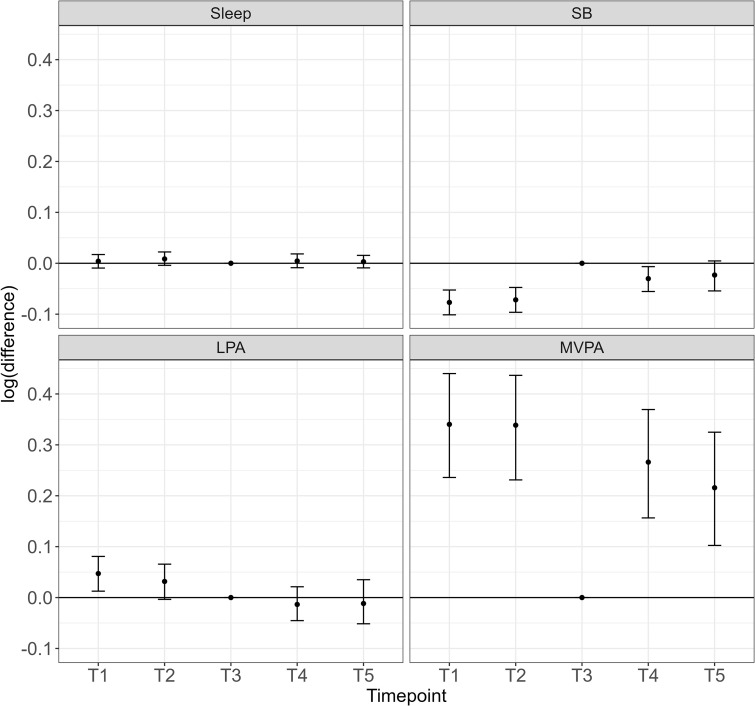
Estimated log-ratio difference in compositional parts for each timepoint when compared to timepoint 3 (school holidays). Note: values above the horizontal line indicate relatively higher proportions of that component during the in-school timepoint compared to the school-holiday period; values below the horizontal line indicate relatively lower proportions of that component during the in-school timepoint compared to the school-holiday period.

We can see that Timepoint 3 during the school-holiday period appears to have the largest differences, particularly in relation to the time spent in SB and MVPA. It is also noteworthy that despite timepoints 3,4 and 5 being significantly and positively associated with $z_{\text{1}}$ (Table 3), suggesting more sleep across these timepoints, we can see that the proportion of sleep is relatively stable across all timepoints, and in fact estimated to be lower in timepoints T3 and T5 than at baseline. The seemingly contradictory results are due to the way $z_{\text{1}}$ must be interpreted. As mentioned in Section 1.1, the first pivot coordinate represents the dominance of sleep relative to the geometric mean of SB, LPA and MVPA. Despite sleep remaining relatively stable across all timepoints, there is a significant change in $z_{\text{1}}$ across timepoints due to the change in the sub-composition of the remaining behaviours (increased contribution of SB and decreased MVPA). This demonstrates why the CMRLMM is preferred to the previously used method of trying to draw inferences about time spent in each behaviour from pivot coordinates in univariate models as has been done previously [34,36]. In order to estimate differences between timepoints we can use the approach suggested by Martín Fernández, Daunis-i-Estadella [58] to determine group differences. Here, we use the fixed effects of the model to calculate the log-ratio difference in the predicted movement-behaviour compositions between timepoint 3 and other timepoints with bootstrapped percentile intervals as shown in Fig 3.

These findings suggest that children’s movement behaviour compositions change as they age, with distinct differences evident during the school-holiday period. These changes are characterised by lower contributions of MVPA and higher contributions of SB. Given the unfavourable associations reported between reallocating time away from MVPA to SB [1], this indicates the school holiday period may be a key intervention point for future public health initiatives.

## 4. Discussion

Key strengths of the CMRLMM outlined in this paper is its ability to include all daily time-use components as dependent variables in a single analytical model, within a multi-level framework. A multi-level framework is relevant for many applications where compositional outcomes are required in epidemiological and behavioural sciences, including both observational and experimental study designs. For example, observational cohort studies that investigate how movement-behaviour compositions change longitudinally over time with repeated measurements on participants (as in our example) or experimental studies with repeated measurements such as randomised-control trials that aim to evaluate compositional effects of a targeted behaviour-change program. Researchers may also be interested in investigating how activity compositions differ among different groups of participants in clustered cross-sectional study designs where participants are sampled within higher-level units such as schools, health-care providers, or worksites. For example, how activity compositions differ amongst different occupational groups when workers are sampled within worksites [59]. In each case, the model’s multilevel structure allows random effects to be defined at relevant grouping levels (e.g., participant, school, or site), thereby accounting for intra-cluster correlation while preserving the compositional dependency between behaviours. This flexibility supports consistent, compositionally valid inference across a wide variety of research designs.

A key benefit of the CMRLMM is that it provides consistent results regardless of how the compositional time-use behaviours are ordered in the model. The CoDA log-ratio transformation overcomes the issue of perfect multi-collinearity between the time-use components, and the variable stacking procedure outlined in this paper enables all the log ratios to be considered as dependent variables simultaneously. Another strength of the analytical pipeline presented in this paper is the interpretability of the model log-ratio coefficients in the original compositional units (minutes/day) via a back-transformation. As we have demonstrated, specific research questions regarding meaningful differences in time use (e.g., comparing children’s time use during school terms to the holidays) can be explored by post-hoc comparisons, and hypotheses via bootstrapping.

There are limitations to the CMRLMM which should be considered. First, the CoDA olr transformation cannot be implemented if there are zero values in any of the time-use components, as the logarithm of zero is undefined. However, this is true of all CoDA techniques, and there are published methods for dealing with zero values in compositional variables, which are beyond the scope of this paper. In terms of the implementation of the CMRLMM in the R environment, it should be considered that these models can have long run times, and convergence issues can arise. However, these issues can be addressed by following recommended troubleshooting procedures (e.g., rescaling and centring continuous variables, trying various optimisers, increasing the maximum number of iterations) [53]. High performance computers may also reduce runtime when fitting models, particularly when modelling compositions with many parts, and parallel processing will drastically reduce runtime when conducting bootstrap inference. Importantly, the CRLMM expressed in compositional coefficients is not more complicated than univariate response models used previously, so the benefits justify the greater computational complexity.

The use of CoDA in time-use epidemiology has grown at a rapid rate in recent years, leading to a paradigm shift in the way that people view the relationship between time use and health. For example, the acceptance that all time-use behaviours are interrelated and jointly contribute to health has led to various countries adopting integrated 24-hour movement behaviour guidelines that promote an optimal mix of these behaviours for different age groups [60–62]. Similarly, multi-component intervention studies now accept the co-dependent nature of time use and aim to simultaneously change time spent in multiple behaviours [7]. Our proposed CMRLMM provides the tools required to allow researchers to better understand how time use changes longitudinally due to natural interventions (such as in the example given in this paper), or in experimental designs. The CMRLMM approach will also support analyses that explore how different personal and sociodemographic factors may be related to different time reallocation patterns. This knowledge will allow more targeted public health initiatives that may aim to block unfavourable reallocations (such as from MVPA to SB as in the current example), or alternatively nudging people to make favourable reallocations.

## Supporting information

S1 File MRLMM-for-CoDa_example. (PDF)

S2 File MRLMM-for-CoDa_modelcomparison. (PDF)

S3 File Mathematical background. (PDF)
